# The Role of Oxidative Stress in Hypomagnetic Field Effects

**DOI:** 10.3390/antiox13081017

**Published:** 2024-08-21

**Authors:** Lanxiang Tian, Yukai Luo, Jie Ren, Chenchen Zhao

**Affiliations:** 1 Key Laboratory of Earth and Planetary Physics, Institute of Geology and Geophysics, Chinese Academy of Sciences, Beijing 100029, China; luoyukai19@mails.ucas.ac.cn (Y.L.); renjie212@mails.ucas.ac.cn (J.R.); zhaochenchen23@mails.ucas.ac.cn (C.Z.); 2 College of Earth and Planetary Sciences, University of Chinese Academy of Sciences, Beijing 100049, China; 3 Beijing National Observatory of Space Environment, Institute of Geology and Geophysics, Chinese Academy of Sciences, Beijing 100029, China

**Keywords:** hypomagnetic field, oxidative stress, reactive oxygen species, signaling pathways, mitochondria

## Abstract

The geomagnetic field (GMF) is crucial for the survival and evolution of life on Earth. The weakening of the GMF, known as the hypomagnetic field (HMF), significantly affects various aspects of life on Earth. HMF has become a potential health risk for future deep space exploration. Oxidative stress is directly involved in the biological effects of HMF on animals or cells. Oxidative stress occurs when there is an imbalance favoring oxidants over antioxidants, resulting in cellular damage. Oxidative stress is a double-edged sword, depending on the degree of deviation from homeostasis. In this review, we summarize the important experimental findings from animal and cell studies on HMF exposure affecting intracellular reactive oxygen species (ROS), as well as the accompanying many physiological abnormalities, such as cognitive dysfunction, the imbalance of gut microbiota homeostasis, mood disorders, and osteoporosis. We discuss new insights into the molecular mechanisms underlying these HMF effects in the context of the signaling pathways related to ROS. Among them, mitochondria are considered to be the main organelles that respond to HMF-induced stress by regulating metabolism and ROS production in cells. In order to unravel the molecular mechanisms of HMF action, future studies need to consider the upstream and downstream pathways associated with ROS.

## 1. Introduction

The geomagnetic field (GMF) is crucial for the survival and evolution of life on Earth because it protects against atmospheric escape, solar wind, and cosmic rays. The elimination of the GMF, known as the hypomagnetic field (HMF), poses risks for astronauts during deep space exploration, as well as for all life during geomagnetic reversals. Paleomagnetic studies have indicated that during reversals, the dipole field strength can decrease by up to 90% at Earth’s surface. This weakens the protection of the GMF, leading to increased radiation on the surface of Earth, exposing all life to the HMF and a strong radiation environment for thousands of years [1,2,3]. The change in Earth’s environment has a profound impact on the evolution and survival of living organisms. Therefore, the role of the GMF on life is composed of two different scales: The GMF’s impact on the origins and evolution of life on Earth and its influence on living organisms’ physiology and behaviors. Although the evolutionary effect of GMF reversals on life is still not fully understood, major variations in the GMF correlate with major geological and biological processes [4,5]. As we look towards future space exploration, astronauts will encounter extremely weak magnetic field conditions, such as in interstellar space (2–8 nT), on the Moon (9–300 nT), and on Mars (10 nT–5 µT) [6,7,8]. These weak magnetic fields could have a negative impact on astronauts’ health. In this article, we focus on the influence of the elimination of GMF on various aspects of animals’ health. Numerous studies have shown that HMF exposure causes many adverse biological effects at both a cellular and organism levels, with changes in the intracellular levels of reactive oxygen species (ROS) often occurring in response to HMF exposure [9,10,11]. 

ROS belong to a large group of oxidants derived from molecular oxygen [12,13]. Oxidative stress occurs when there is an imbalance favoring oxidants over antioxidants, leading to redox signaling disruption and/or molecular damage. In recent years, with a better understanding of the roles of different ROS in cells, “oxidative stress” has come to encompass two meanings: elevated levels of ROS cause molecular damage, known as ‘oxidative stress’, and physiological levels of ROS play key roles in redox signaling through post-translational modifications, known as ‘oxidative eustress’ [14,15]. ROS are essential for cellular signaling and response to stress, which contribute to both physiological and pathological conditions. Moderate ROS levels serve as signaling molecules and are essential for cellular functions and various pathologies [16]. ROS assist the host in combating micro-organisms and also participate in intermicrobial competition. For instance, mouse peritoneal macrophages can be stimulated to release H_2_O_2_ against micro-organisms into the extracellular medium [17], while excess ROS levels incur damage to DNA, protein, or lipids, widely associated with dysfunction and disease. The interaction of ROS with nitrogenous bases and deoxyribose results in significant oxidative DNA damage, leading to mutations, carcinogenesis, apoptosis, necrosis, and hereditary diseases [18]. Most cancers are believed to have a direct connection to ROS-induced mitochondrial dysfunction. This direct relationship between ROS and cancer has also been identified in other conditions, such as Alzheimer’s and Type 2 diabetes [19]. Research indicates that HMFs have a significant impact on cellular ROS levels, altering physiological and biological processes in organisms [20,21]. Signaling pathways associated with ROS regulation may be linked to many biological effects caused by HMF exposure. Here, we outline the upstream and downstream signaling pathways of ROS and propose the possible molecular mechanisms underlying the HMF action.

## 2. Types of ROS

ROS encompass molecules derived from O_2_, including superoxide (O_2_^•−^), hydrogen peroxide (H_2_O_2_), and hydroxyl radical (^•^OH). Superoxide dismutases (SODs) catalyze the dismutation of O_2_^•−^ to H_2_O_2_. H_2_O_2_ is reduced by many kinds of enzymes (antioxidants), including catalase, peroxiredoxins (PRDXs), glutathione peroxidases (GPXs), and other peroxidases (cytochrome c). Catalase is one of the key antioxidant enzymes that destroys cellular hydrogen peroxide to produce water and oxygen to relieve oxidative stress [22]. O_2_^•−^ can damage various cellular components and indirectly affect signaling pathways [18,23]. H_2_O_2_ serves as a signaling molecule through the reversible oxidation of cysteine residues within proteins, which is involved in redox signaling at specific concentrations. For example, the thiolate anion of cysteine residues is oxidized to sulfenic form (Cys-SOH) by low levels of H_2_O_2_, which alters the function of protein during redox signaling [24,25], while higher levels of H_2_O_2_ further oxidize thiolate anions to sulfinic (SO_2_H) or sulfonic (SO_3_H) species to cause permanent protein damage [18]. The most harmful effect of H_2_O_2_ is its reaction with transition metals such as ferrous iron (Fe^2+^) to produce hydroxyl radicals (^•^OH), known as the Fenton reaction. ^•^OH is a powerful oxidant that indiscriminately oxidizes lipids, proteins, and DNA, causing significant damage and genomic instability. In addition, other oxygen-containing free radicals can also cause oxidation of essential cellular components, including peroxynitrite, lipid hydroperoxides (LOOH), alkoxyl radical (RO^•^), peroxyl radical (ROO^•^), sulfate radical (SO_4_^•−^), and Fenton reaction intermediates [26,27]. Here, we mainly discuss two types of ROS involved in the biological effects of HMF: O_2_^•−^ and H_2_O_2_.

## 3. ROS Homeostasis

ROS levels are strictly controlled by multiple ROS-generating and ROS-eliminating systems, which actively maintain the intracellular redox state in cells. Production of O_2_^•−^ is primarily from the mitochondrial respiratory chain and the NADPH oxidases (NOXs) in cells. NOXs are transmembrane enzymes responsible for the production of O_2_^•−^ via electron transfer across membranes from NAD(P)H to molecular oxygen [28]. NOXs produce superoxide primarily on the intracellular side of membranes. The human NOXs family consists of Nox1–5, Duox1, and Duox2. The classic activity control of Nox enzymes is exerted by calcium or protein–protein interactions and post-translational modifications (PTMs). Phosphorylation, acetylation, methylation, and glycosylation are the most studied PTMs in cells. The activities of Nox5 and the Duox enzymes are calcium-dependent, and the activity controls of Nox1, Nox2, and Nox3 are regulated through interactions with the cytosolic proteins, such as the small GTPase Rac [29]. Interactions between different oxidases or oxidase systems play key roles in oxidative stress. Activation of NOX induces activation of downstream secondary oxidase systems, including uncoupled endothelial nitric oxide synthase and xanthine oxidase [30]. 

Mitochondrial electron transport chain (ETC) complexes I and III are the main sites of O_2_^•−^ production where the leaking O_2_ is reduced to O_2_^•−^. The NADH and FADH_2_ donate electrons to the ETC. Thus, the ratio of NADH to NAD^+^ regulates O_2_^•−^ production [22,31]. Therefore, there is a close relationship between mitochondrial oxidative phosphorylation (OXPHOS) and mitochondrial ROS (mtROS) generated by the ETC. The mtROS production depends on the metabolic state of mitochondria in cells [32]. In response to the electron transport, protons (H^+^) are pumped from the matrix into the intermembrane space to form mitochondrial membrane potential (ΔΨ_m_). ATP synthase uses the energy of proton gradient to produce ATP. However, uncoupling proteins (UCPs) break the perfect coupling between proton gradient and ATP synthase, mediating a small amount of H^+^ to flow into the matrix to reduce ATP synthesis. Therefore, UCPs are able to reduce the efficiency of OXPHOS, thereby reducing ROS production [33]. Other than mitochondria and NOXs, other sources of ROS production include xanthine oxidase (XO) and cyclooxygenases (COXs) [34].

ROS are counterbalanced by antioxidant networks, which modulate ROS levels to allow their physiological roles. ROS-eliminating systems and antioxidants include GPXs, thioredoxin peroxidases (TRXPs), SODs, PRDXs, glutathione (GSH), thioredoxin 2 (TRX2), glutaredoxin 2 (GRX2), ascorbic acid, tocopherol, vitamin E, and carotene [35]. For example, SODs convert O_2_^•−^ to H_2_O_2_, which can then be converted by catalase to harmless H_2_O [32,36].

## 4. Changes in Cellular ROS Levels Caused by HMF

The effects of HMF on living organisms are diverse, including different effects ranging from cells, tissues, and organs to organisms. For instance, HMF has significant negative effects on early development, circadian rhythms, and the central nervous system in animals [9,37]. However, the mechanisms underlying these effects are still not well understood. Here, we summarize published studies on the effects of HMFs on ROS within animal cells or organisms (Table 1), excluding studies on plants or micro-organisms. HMF generally reduced H_2_O_2_ levels within cells, accompanied by changes in cell behavior and gene expression in in vitro studies (Table 1). HMF in the range of 0.2–0.5 μT inhibited the proliferation and *eNOS* expression in endothelial cells [38], while HMFs (0.5–2 μT) suppressed H_2_O_2_ production in cancer cells and bovine pulmonary artery endothelial cells (PAEC). This effect can be inhibited by antioxidants like catalase and SOD mimetic MnTBAP [39]. HMF (<0.2 μT) reduced H_2_O_2_ levels in human neuroblastoma cells by inhibiting the activity of CuZn-SOD, and the enhanced cell proliferation caused by HMF can be remedied by additional H_2_O_2_ supplementation [40]. HMF (20 nT) also reduced ROS production in mice peritoneal neutrophils by affecting NOX activity and mitochondrial ETC [41,42]. However, an in vitro study of mouse skeletal muscle cells showed that HMF (<3 μT) could cause an increase in its ROS levels, leading to a decrease in cell function [43]. 

As shown in Table 2, we have found that HMF (0.29 ± 0.01 μT) reduced endogenous ROS in adult hippocampal neural stem cells, further affecting cognitive function of the hippocampus in mice [11]. ROS levels are tightly controlled by an inducible antioxidant program that responds to cellular stressors. Further, in vivo research on other hippocampal cells in the mouse hippocampus revealed that HMF (31.9 ± 4.5 nT) significantly increased its ROS levels by decreasing the expression of antioxidant genes, which may cause oxidative stress damage to the overall hippocampus and further affect anxiety and cognitive behavior in mice [44,45]. HMF can cause bone loss in mammals. For instance, HMF (<300 nT) promotes additional bone loss in the mouse femur during mechanical unloading, likely due to iron overload, exacerbating oxidative stress and thereby inhibiting osteoblast activity [46,47]. Other research showed that under particular conditions of oxidative stress, HMF (0.192 μT) could disrupt the functional state of erythrocytes and promote cell death in rats [48]. In addition, neuroinflammation regulates stem cell niches, particularly neural stem/progenitor cells in mammals. We have found that HMF (31.1 ± 2.0 nT) may also cause neuroinflammation in the hippocampus of mice, manifested by the activation of microglia and upregulation of GFAP expression in astrocytes [49]. This may be closely related to the increase in ROS in hippocampal cells [44,45]. In the biological effects of HMF, changes in ROS levels in cells are often accompanied by changes in cell number, proliferation, and survival; changes in the expression level of antioxidant genes; and even behavioral abnormalities, such as a decline in cognitive and orientation abilities, in animals (Table 1 and Table 2). The different results of HMF affecting ROS levels may be related to the differences in magnetic strength, duration of exposure, cell type, or method of HMF generation.

## 5. ROS-Mediated Signaling Pathways and Potential Links to HMF Effects 

**Figure 1:**
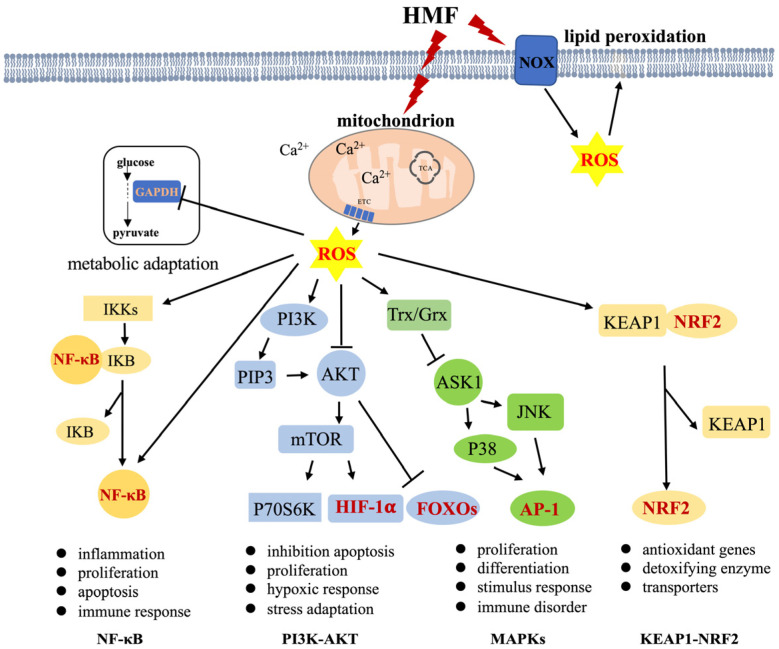
ROS-regulated signaling pathways. ROS act as second messengers to regulate four main signaling pathways, controlling a variety of biological processes in cells. Mitochondria and NOXs are the two main sources of ROS generation (black arrows: activation; black T-arrows: inhibition). Dark red font represents the transcription factors. TCA: tricarboxylic acid cycle, ETC: electron transport chain, AP-1: activator protein-1.

ROS have beneficial or harmful effects depending on their local concentration in a cell. Suitable levels of ROS are involved in numerous crucial physiological processes, including proliferation, differentiation, metabolic adaptation, and the regulation of adaptive and innate immunity. However, excess ROS can be harmful to maintaining cellular redox homeostasis, inevitably causing damage to macromolecules or inducing cell death. which contributes to the development of many diseases, such as hypertension, cardiovascular diseases, diabetes, cancer, neurodegeneration, and aging [15,18]. The possible signaling pathways mediated by ROS are nuclear factor-κB (NF-κB) and phosphoinositide-3-kinase (PI3K)-Akt pathways, mitogen-activated protein kinase (MAPK)s, and Keap1-Nrf2 signaling pathways, which regulate various physiological/pathological functions, including cell proliferation and differentiation, growth and apoptosis, and the response to external stress and inflammation (Figure 1) [53,54,55,56]. Moderate elevations of H_2_O_2_ mediate GAPDH oxidation in glycolysis, and the inactivation of GAPDH stimulates the oxidative pentose phosphate pathway of glucose. ROS as pleiotropic physiological signaling agents are thoroughly summarized in two recent papers [15,57]. The following are brief summaries concerning signaling pathways mediated by ROS (Figure 1).

(1) The transcription factor NF-κB regulates the expression of various inflammatory genes and mediates the inflammatory response to stressful stimuli. The activation of NF-κB involves two major signaling pathways — the canonical and noncanonical pathways—which are important for regulating immune and inflammatory responses [58]. In addition, NF-κB plays a critical role in regulating the survival, activation, and differentiation of innate immune cells and inflammatory T cells. NF-κB activity influences ROS levels by regulating the expression of antioxidant (SOD2) or pro-ROS enzymes, while ROS have various inhibitory or stimulatory roles in NF-κB signaling in a context-dependent manner [59,60]. ROS has been shown to regulate the upstream NF-κB activating pathways; for example, ROS activates NF-κB through alternative IκBα phosphorylation in IKKs [61], and H_2_O_2_ markedly decreased the ability of TNF to induce IKK activity, resulting in the prevention of I-κB degradation and NF-κB activation [62]. Meanwhile, sustained oxidative stress may lead to the inactivation of the proteasome and subsequently inhibit NF-κB activation by impeding the degradation of I-κB [63]. mtROS are intermediates that trigger inflammatory signaling cascades, which enable proinflammatory signaling by inducing the disulfide linkage of NF-κB essential modulator (NEMO). This bond is essential for activating the NF-κB and MAPK pathways and leading to proinflammatory cytokine secretion [64]. Both mtDNA and mtROS from dysfunctional mitochondria can drive IL-1β and IL-18 secretion as a consequence of inflammasome signaling [54]. Direct or indirect oxidation of NF-κB heterodimers by ROS inhibits its DNA binding ability or activates the interaction of RelA with CBP/300 [65,66]. NF-κB also regulates some enzymes that promote the production of ROS in immune cells [67,68,69]. Moreover, NF-κB signaling crosstalk influences various signaling pathways, including STAT3, AP1, interferon regulatory factors, NRF2, Notch, WNT–β-catenin, and p53 [59,70,71,72]. Kim et al. reported that H_2_O_2_ modulates IKK-dependent NF-kB activation by promoting the redox-sensitive activation of the PI3K/PTEN/Akt and NIK/IKK pathways [73]. There is an extremely close relationship between the NF-κB signaling pathway, ROS-generating enzymes, and antioxidant enzymes in inflammation-related injuries and diseases. The activation of NF-κB is a key link in the inflammatory response, which mediates IL-1 β, TNF-α, and iNOS expression [74,75,76]. Inhibiting the activation of NOXs can inhibit NF-κB activation to alleviate inflammation [77,78,79,80]. 

(2) PI3K/AKT pathway is downstream of the EGFR/HER family and upstream of mTOR. It regulates various cellular activities, such as cell cycle progression and cancer onset. PI3K/AKT signaling can be regulated by ROS in a concentration-dependent manner. Chen et al. showed that excess ROS hinder the PI3K/AKT pathway, causing apoptosis and inflammation in corneal epithelial cells (CECs). However, at the appropriate level, ROS activate PI3K/AKT signaling, inhibiting apoptosis and promoting the proliferation of CECs [81]. For instance, ROS can inactivate PTEN by oxidizing the active site cysteine residues, which upregulates the PI3K/AKT pathway [82]. These are responsible for negatively regulating PIP3 synthesis, thereby inhibiting Akt activation by oxidizing cysteine residues inside the active center [83,84]. In addition, hypoxia-inducible factor 1α (HIF1α) can regulate cell adaptation to hypoxia and other stimulators. Tian et al. showed that the PI3K/AKT pathway can upregulate the expression of HIF1α in breast cancer cells [85]. Forkhead box O (FOXO) members in humans are modulated by the PI3K/AKT pathway, which predominately responds to stress conditions [86,87]. 

(3) The MAPK pathways are important bridges in the switch from extracellular signals to intracellular responses, regulating processes like cell proliferation, differentiation, survival, cancer dissemination, and resistance to drug therapy. There are three main MAPK cascades: extracellular-signal-regulated kinase 1 and 2 (ERK1/2), c-Jun N-terminal kinases (JNK), and p38 kinase (p38). MAPK, MAPKK, and MAP3K (also known as ASK1) are classic protein kinases in each cascade [88]. Reduced thioredoxin (Trx) is a key negative regulator of ASK1 kinase activity. Trx contains Cys32 and Cys35 cysteine residues in its active site. In response to oxidative stress, H_2_O_2_ oxidizes these cysteine residues, leading to the release of Trx, which is required for the kinase activity of ASK1. TNF receptor-associated factor (TRAF)2 and TRAF6 are then recruited to the ASK1 signalosome, resulting in the activation of the JNK and p38 MAPK pathways [89,90]. 

(4) NRF2-KEAP1 plays a crucial role in maintaining redox homeostasis and metabolism in cells. NRF2 is an important transcription factor for the expression of antioxidant proteins, protecting cells from oxidative damage. Meanwhile, KEAP1 serves as a negative regulator of NRF2 by binding to the Neh2 domain of NRF2 [91]. Evidence indicates that under stress conditions, ROS directly oxidizes KEAP1-cystines, leading to its dissociation and NRF2 translocation to the nucleus to regulate the genes of antioxidants [89,91].

These pathways regulate various downstream physiological and pathological functions. ROS, especially H_2_O_2_, can act as a second messenger or damaging oxidative stress, triggering some key signaling pathways through interaction with critical signaling molecules to regulate many downstream cascade events. This results in various physiological effects and behavioral abnormalities, such as cognitive dysfunction and anxiety in animals exposed to HMF. We suggest that understanding the relationship between these signaling pathways and HMF exposure is key to uncovering the molecular mechanisms underlying these biological effects. 

Research has shown that HMF affects the mitochondria and NOXs, which are responsible for producing ROS. HMF exposure also leads to reduced mitochondrial ΔΨ_m_, decreased ATP production and mitochondria activity, elevated ROS levels, and disrupted Ca^2+^ balance in cells (Table 1 and Figure 1). Under oxidative stress, ROS, especially H_2_O_2_, activate signaling pathways to initiate biological processes, while high levels of ROS result in damage to DNA, protein, or lipids. These effects are closely linked to cellular mitochondrial dysfunction [43,49,92,93]. PGC-1α, a regulator of mitochondrial biogenesis and an important inducer of antioxidant gene expression during oxidative stress, has been found to significantly reduce in expression levels due to HMF exposure, with a decrease in the number of active mitochondria in the hippocampus [45]. Low PGC-1α expression inhibits the induction of various ROS-detoxifying enzymes, resulting in increased ROS levels [94]. As a result, we propose that mitochondria are considered to be the primary organelles responding to HMF-induced stress by regulating the metabolism and ROS production in cells. We speculate that the shift between glycolysis and oxidative phosphorylation is a response to HMF. Although it is still unclear how HMF affects ROS production by regulating upstream signals, we speculate that certain molecules/ions (e.g., Ca^2+^) within cells or organelles may sense HMF as an external environmental stimulus, leading to changes in NOXs activity or mitochondrial dynamics and function, ultimately affecting cellular ROS levels. ROS serves as a second messenger or causes oxidative stress, triggering key signaling pathways or oxidative damage to macromolecules, resulting in a series of downstream cascades (Figure 1). However, the question of which biological effects caused by HMF are regulated by ROS-regulated signaling pathways still needs experimental verification. Additionally, *N*-acetyl cysteine (NAC) or γ-glutamylcysteine (γGC) can be attempted to relieve oxidative stress and mitigate the negative effects of HMF. For example, NF-κB can be activated by high levels of H_2_O_2_, and this activation is blocked by treating the cells with the anti-oxidant NAC [95,96] and γGC, as a precursor to GSH, which may increase GSH levels in healthy subjects, suggesting its potential to detoxify H_2_O_2_ in a GPX1-dependent manner [97,98].

## 6. Conclusions

HMF has been found to cause cognitive dysfunction, anxiety-related behaviors, circadian disorders, gut dysbiosis, reproductive and developmental abnormalities, and osteoporosis in animals [37]. These functional abnormalities in animals may be linked to cellular-level effects caused by HMF. For example, HMF inhibited adult hippocampal neurogenesis, led to high ROS levels and inflammation in the hippocampus, and altered the concentrations of melatonin and norepinephrine in animals. These effects may collectively contribute to cognitive impairment in the animals. HMF also leads to osteoporosis by inhibiting osteoblast differentiation and mineralization. ROS may play key roles in the regulatory mechanisms behind these HMF effects through one or more signaling pathways. However, the question of which biological effects caused by HMF are regulated by ROS-regulated signaling pathways needs to be confirmed in future studies. Mitochondria are considered to be the primary organelles responding to HMF-induced stress. Changes in NOX activity and mitochondrial dynamics and function caused by HMF affect the cellular ROS level. H_2_O_2_ acts as a second messenger molecule or oxidative stress, leading to a series of downstream cascades. To reveal the molecular mechanism of HMF effects, the upstream and downstream pathways related to ROS need to be considered in future studies. Therapeutic methods targeting oxidative stress and mitochondrial dysfunction could be developed to mitigate the negative effects of HMF in the future. 

## Figures and Tables

**Table 1:** ROS changes caused by HMF at cellular level.

Cell Lines	Magnetic Intensity	Exposure Time	ELF-EMF in HMF/GMF	Changes in ROS Level	Other Biological Effects	Refs.
Primary mouse skeletal muscle cells	HMF < 3 µT	3 days 1 h for isolated mitochondria	105.1 ± 9.2 nT (52–66 Hz)/763.1 ± 79.1 nT (134–178 Hz) (Alternating current magnetic field)	low glucose consumption high ADP/ATP ratio low membrane potential (ΔΨm) ROS level increased	HMF-exposed cells showed a decline in cell viability of primary mouse skeletal muscle cells relative to GMF control.	[43]
Fibrosarcoma HT1080/ pancreatic AsPC-1 cancer cells, primary endothelial cells	0.5–2 µT (In the µ-metal cylinder)	6, 12, and 24 h	1620 nT/6080 nT (60 Hz)	H_2_O_2_ level decreased in HT1080 cells H_2_O_2_ level decreased in endothelial cells H_2_O_2_ in pancreatic cells not changed		[39]
Human umbilical vein endothelial cells	0.2–0.5 µT	24 h per day for 3 days		NOS expression decreased * NO concentration not changed	HMF decreased the endothelial cell number and did not change VEGF gene expression.	[38]
Human neuroblastoma cell line SH-SY5Y	<0.2 μT		22.5 ± 0.0 nT/77.4 ± 1.2 nT (50 Hz)	H_2_O_2_ level decreased CuZn-SOD decreased	HMF inhibited the activity of CuZn-SOD by accelerating protein denaturation and enhancing its aggregation in vitro.	[40]

* This result obtained in comparison to the group treated with 120 μT static fields.

**Table 2:** ROS changes caused by HMF at organism level.

Animal	Tissue/Organ	Magnetic Intensity	Exposure Time	ELF-EMF in HMF/GMF	Changes in ROS Level	Other Biological Effects	Refs.
C57BL/6J male mice	Hippocampal cells	~32 nT	4, 6, 8 weeks	0.5 nT/1.2 nT (50 Hz)	O_2_^•−^ level increased at 8th week of exposure	The number of active mitochondria was decreased at 6-week HMF; The expression level of the PGC-1α gene was markedly reduced in the hippocampus at 8-week HMF.	[45]
Black Garden Ant		40–50 nT	14 days		H_2_O_2_ levels with a 45% reduction Cytoplasmic SOD increased Glutathione reductase decreased	HMF increased the time required for ant workers to find food and return to the nest; There is a general drop in brain biogenic amine but not melatonin.	[50]
C57BL/6J male mice	DG and CA regions of hippocampus	~32 nT	8 weeks	0.83 nT/0.06 nT (50 Hz)	O_2_^•−^ levels increased Expression levels of *Nox4*, *Epx*, *Krt1*, and *Nos2* increased Expression levels of *Gpx3* and *Hspa1a* decreased	8-week HMF led to cognitive impairments in mice.	[44]
C57BL/6J male mice	epithelial layers of colon	~31 nT	8, 12 weeks		O_2_^•−^ levels increased	8-week HMF decreased the concentrations of short-chain fatty acids and promoted colonic cell proliferation.	[51]
C57BL/6J male mice	aNSCs in hippocampus	~290 nT	8 weeks		O_2_^•−^ levels decreased in aNSCs in vivo	8-week HMF caused significant impairments of adult hippocampal neurogenesis and hippocampus-dependent learning ability in mice.	[11]
C57BL/6J male mice	muscle samples	<500 nT	30 days	230 nT/690 nT (120 Hz) (Alternative magnetic fields)	The number of mitochondria decreased Morphology of mitochondria changed	One-month HMF significantly reduced the endurance of the HMF-exposed mice and decreased the citric acid level in skeletal muscles.	[52]
CD-1 adult male mice	mice peritoneal neutrophils	20 nT		GMF: 15–50 nT (50 Hz)	ROS production decreased NADPH oxidase activity decreased Mitochondrial ETC decreased		[41,42]

DG: Dentate Gyrus, CA: Cornu Ammonis, aNSCs: adult neural stem/progenitor cell.
